# Accelerators/decelerators of achieving universal access to sexual and reproductive health services: a case study of Iranian health system

**DOI:** 10.1186/1472-6963-13-241

**Published:** 2013-07-01

**Authors:** Nahid Akbari, Ali Ramezankhani, Mehrnoosh Pazargadi

**Affiliations:** 1 Department of Midwifery and Reproductive Health, Nursing and Midwifery School, Shahid Beheshti University of Medical Science, Tehran, Iran; 2 Department of Public Health, Shahid Beheshti University of Medical Science, Tehran, Iran; 3 Department of Nursing, School of Nursing and Midwifery, Shahid Beheshti University of Medical Science, Tehran, Iran

**Keywords:** Iran, Health system, Sexual and reproductive health, Universal access, Millennium development goal

## Abstract

**Background:**

At the 1994 International Conference on Population and Development (ICPD), held in Cairo, the global community agreed to the goal of achieving universal access to sexual and reproductive health (SRH) and rights by 2015. This research explores the accelerators and decelerators of achieving universal access to the sexual and reproductive health targets and accordingly makes some suggestions.

**Method:**

We have critically reviewed the latest national reports and extracted the background data on each SRH indicator. The key stakeholders, both national and international, were visited and interviewed at two sites. A total of 55 in-depth interviews were conducted with religious leaders, policy-makers, senior managers, senior academics, and health care managers. Six focus-group discussions were also held among health care providers. The study was qualitative in nature.

**Results:**

Obstacles on the road to achieving universal access to SRH can be viewed from two perspectives. One gap exists between current achievements and the targets. The other gap arises due to age, marital status, and residency status. The most recently observed trends in the indicators of the universal access to SRH shows that the achievements in the “unmet need for family planning” have been poor. Unmet need for family planning could directly be translated to unwanted pregnancies and unwanted childbirths; the former calls for sexual education to underserved people, including adolescents; and the latter calls for access to safe abortion. Local religious leaders have not actively attended international goal-setting programs. Therefore, they usually do not presume a positive attitude towards these goals. Such negative attitudes seem to be the most important factors hindering the progress towards universal access to SRH. Lack of international donors to fund for SRH programs is also another barrier. In national levels both state and the society are interactively playing their roles. We have used a cascade model for presenting the barriers at the state levels from the strategic planning to implementation. Social factors are to be considered as a background for other factors at all stages.

**Conclusion:**

Accelerating universal access to SRH requires adequate funding, firm political commitment, creative programming, and the involvement of diverse actors, including faith-based, civil society, and private sector partners.

## Background

### The millennium development goals

At the 1994 International Conference on Population and Development (ICPD), held in Cairo, the global community agreed to the goal of achieving universal access to sexual and reproductive health (sexual and reproductive health) and rights by 2015. Since 2005, this target has also been regarded as pre-requisite for achieving the Millennium Development Goals. The goals encompass improving maternal health, reducing poverty and gender inequality, and combating HIV/AIDS and other diseases. As such, the target of universal access to sexual and reproductive health services is an important objective for the health system in various countries. The ICPD Program of Action called for paying more attention to people–centered approach in health systems [1-3].

Access to a range of sexual and reproductive health services that safely and effectively satisfy both men’s and women’s needs is considered as human right. A well-functioning health system would provide right-based interventions to improve access to sexual and reproductive health information and services [1-3]. *sexual and reproductive health* has been recognized to be “**A Matter of Life and Death**” [4], nonetheless, there continues to be “**A Sorry Story:***… services are absent or of poor quality and underused in many countries because discussion of issues such as sexual intercourse and sexuality make people feel uncomfortable. The increasing influence of conservative political, religious, and cultural forces around the world threatens to undermine progress made since 1994, and arguably provides the best example of the detrimental intrusion of politics into public health…*[4,5].” Addressing the 5th Asian and Pacific Population Conference (Bangkok: UNESCAP, December 2002), Kofi Annan, United Nations Secretary-General stated that “*the Millennium Development Goals, particularly the eradication of poverty and hunger, cannot be achieved if questions of population and reproductive health are not squarely addressed. And that means stronger efforts to promote women’s rights, and greater investment in education and health, including reproductive health and family planning*[6].” After years of pressure from the global community, in 2006 the United Nations General Assembly finally accepted the target of achieving, by 2015, universal access to reproductive health as an additional component of the goal 5 of the MDGs (improved mental health), now known as target 5B.

### Health system of Iran and reproductive health agenda: progress and gaps

#### Progresses

Iran is an upper middle-income country estimated to have a population of 73 974 000 in 2011 [7,8]. According to the World Health Organization’s National Health Account data, Iran spent 6–7.0% of GDP on health [8,9]. One third of the Iranian people are younger than 15 years of age with only 5% over 60 years old. The annual population growth rate in 2008 was 1.2%. Currently, 99% of population are Muslim [10], with 91% being Shiite. Some development indicators have been constantly improving especially in terms of infant mortality rate and life expectancy. Iran’s public health system provides health cares via “Urban Health Care Centers” and “Rural Health Houses” in urban and rural areas, respectively. Currently there are more than 4000 health centers in rural and urban areas [11]. Efforts to remove obstacles on the road to achieving universal access to health care include the ratification of the Social Security law in 1975 [12], establishing the Health Care Network in 1984 with the objective of securing equal and fair access of all Iranian to primary health care services [13], the ratification of health care insurance law in 1994 with the objectives of separating health care service providers from the financiers and covering the entire population by 1999, and finally the ratification of the family physician law in 2004 [14,15]. Health services are now divided into primary health care (PHC) and medical benefit packages, with their related financing systems.

The consequences of the aforementioned measures have been reported previously. For instance, a favorable trend in the maternal mortality rate has been reported and attributable to 5 major reasons including a decreased fertility rate [16-18], later marriages and childbirths [19], improved rural women’s literacy [20-22], better access to emergency health (especially obstetric) services, and broadened maternal and childbirth care via the nation-wide health network. Over the past two decades Iran has experienced the world’s most rapid fertility decline that could be, at least in part, explained by substantial increases in female education [22].

The parameters for abortion have changed. Although abortion is limited to therapeutic indications as defined by the law, in the case of illegal abortions, postoperative care is available in public and private hospitals as part of primary health care [23,24]. Health care providers do not feel compelled to report illegal abortions to authorities and these cases are not prosecuted unless someone records a grievance. Emergency contraception is also available in family planning clinics [24].

#### Gaps

About one third of pregnancies continue to be unwanted [25] and there is much room for induced abortions to be decreased [26-33]. In Iran, the induced abortion is generally considered to be illegal [34]. The few exceptions are situations in which continuing pregnancy is deemed to impose a risk to mother and a few known fetal anomalies [24]. Despite the dramatic decline in fertility in recent decades, limited access to legal abortion continues to lead many women whose pregnancies are unwanted or mistimed to undergo clandestine, unsafe abortions. No official data on the abortion rate in Iran have been collected; however Erfani et al. have used the 2000 Iran Demographic and Health Survey and estimated the total abortion rate to be 0.26 abortions per married woman, and the annual general abortion rate to be 7.5 abortions per 1,000 married women aged 15–49 years. They reported that modern contraceptive usage more effectively (31%) reduced the abortion than did religiosity [35].

### Iran and millennium development goals

Gaps in the Sexual and Reproductive Health in Iran could be looked upon from two perspectives: first, the gap between the current situation and the MDGs and the second, disparities that exist among the Sexual and Reproductive Health status of the different subgroups of the population. Different domains of the Sexual and Reproductive Health have been promoted with different pace and the pattern of growth varies across different subpopulations.

#### Toward achieving goals

Enormous gains in Sexual and Reproductive Health have been achieved by Iran over the last decade, due in part to patience and persistence in dealing with culturally sensitive issues [36].

The shift from one of the fastest growing countries in the world to a country with replacement fertility in less than 20 years has made Iran a unique case to study. The initiation of fertility decline coincided with the establishment of Health Care Network. The network was supposed to implement the Population Control Program by promoting access to family planning services. Afterward, the trends in fertility paralleled the expansion of Health Care Network, which is based on Urban Heal Centers and Rural Health Houses. There are currently more than 31 000 Community Health Workers (Behvarz, behværz) working in more than 19 000 Health Houses in rural areas [37]. The new family planning program has been demonstrated to significantly reduce the relative risk of higher order births. The program effect was dramatically strengthened after passing the new family planning bill in 1993 [38-42]. The effect of program on first births is marginal and not significant. The program has not succeeded in delaying the first birth. There are, however, clear evidences to indicate that the program played a major role in delaying and stopping other births especially third birth and higher. On the other hand, women’s education has been shown to confer a much stronger negative effect than the Population Control Program. Currently, there are several studies in the literature attributing the fertility decline to development and put less emphasis on the role of family planning programs [42,43]. Other contributing factors have been reported to be an increase in late marriages and childbirths, improved rural women’s literacy, better access to emergency health services [44].

Achievements made and challenges encountered by Iran were also discussed in the first (2004) and second (2006) Millennium Development Goals reports [44,45]. The country has reported to have far exceeded the targets laid out at the 1994 International Conference on Population and Development in Cairo. The maternal mortality rate per 100 000 live births dropped sharply from 91 in 1989 to 37.4 deaths in 1997, and then rather slowly to 24.6 in 2005. According to the target defined for this MDG, this indicator would need to further decrease to 18–22 deaths per 100 000 live births by 2015. Thus, it seems that meeting this target within the time remaining until the year 2015 would be feasible. Likewise, the proportion of births assisted by skilled attendants was increased from 70% in 1989 to about 90% in 2000 and 97%, thereafter (institutional delivery is now 96%). The contraceptive usage prevalence rate increased from 49.6% in 1989 up to 73.8% in 2000 [17]. Moreover, the adolescent fertility rate almost halved from 54 children per 1000 women in 1996 to 26.8 children per 1000 women in 2000. Antenatal care coverage has been reported to be 98.3% for at least one visit and 94% for at least 4 visits [46]. Unmet need for family planning varied from 3.6% in Tehran to 31.3% in one of the most disadvantaged provinces named (Sistan-Balouchestan). The average unmet family planning need for the country has reported to be 7.6% [47]. In the developing world, the unmet need for contraception is of utmost importance among adolescents [48].

#### Uneven, patchy distribution of the access to the sexual and reproductive health

There are regional disparities in Sexual and Reproductive Health indicators such as births attended by skilled birth attendants. Significant differences could be observed for certain provinces with regard to maternal mortality. For example, the province of Sistan-Baluchestan, although registering only 6% of total births, has 13% of maternal deaths in the country. Also, while only 2.94% of women ages 15–49 live in Sistan-Baluchestan, 6% of total births take place in this province (high fertility rate). Pregnancy and childbirth therefore carry a higher risk of death in this province in comparison with other areas in the country. A similar situation, but to a lesser extent, exists for the provinces of Kerman, Kohgiluyeh-Buyer Ahmad, Kermanshah, Hormozgan and Hamadan. The opposite is true for the province of Tehran. Although 15% of total births take place in this province, only 6% of maternal deaths happen in Tehran. Although, findings on the maternal deaths in Tehran is likely subject to under-estimation; since a considerable amount of data comes from private sector and other service providers, from whom the collection of data is not easily possible. Furthermore, coverage of modern methods of contraception increased in rural areas from 43.5% in 1993 to 66.9% in 2005, with the highest increases being reported for the rural areas of Zanjan, Ardebil and Kermanshah, with 80, 76.4 and 75.7%, respectively; and the lowest increases for the rural areas of Sistan-Baluchestan (38.6%) and Hormozgan (43.8%) [45,49]. In the Third Country Program (2000–2004), The United Nations Population Fund (UNFPA) is assisting the Government with empowering women and youth and decreasing the illnesses and deaths of mothers and children. In this program disadvantaged, hard-to-reach areas, where health indicators are lower than the national average have been given a particular attention. These areas are also home to two important ethnic communities, the Baluchis and the Kurds, for whom issues related to family planning, are extremely sensitive [36].

### Current study

Despite the abovementioned achievements, there continues to be several serious challenges mandating urgent action [50-53]. Ensuring universal access to sexual and reproductive health targets and rights is essential for achieving many of the MDGs, especially those on maternal health, child survival, HIV and AIDS, and gender equality [48]. The accelerators and decelerators of achieving universal access to sexual and reproductive health in Iran have been scarcely investigated. A major challenge is to deal with unmet family planning needs and adolescent education. Unintended births require consideration of the conditions guiding access to safe abortion. These issues are generally considered as taboo. [52,53]. We aimed, thus, to determine the factors that might have impeded or promoted Iran’s health system achieving public access to sexual and reproductive health services.

## Methods

### Study population

This study aimed at improving our understanding of the challenges being faced by the Iranian health system in terms of the reproductive health agenda. As such, we have taken a qualitative approach, exploring the opinions of key stakeholders and experts in the field. The study adheres to the RATS guidelines on qualitative research. Appropriate interview guides were specifically designed for use in the study and during 2010–2012, 55 key in-depth interviews (KII) and six focus group discussions (FGDs) were carried in Tehran, the capital of Iran. We also conducted web searches and followed related links in order to find key stakeholders (parliament representatives, health committee) for interviewing. This approach also enabled us to review existing literature on stakeholders’ perspectives.

A purposive sample of 54 participants was selected among senior officials from government departments (health, education, and welfare), religious leaders; senior academics, managers of four NGOs and health care providers. The majority of these participants had contributed to the design or implementation of reproductive health and HIV/AIDS programs in Iran. The mean age of the participants was 43 year (ranging between 31 and 58 years); with the mean of working experience being 9 years (ranging between 4 and 31 years).

Participants were interviewed face-to-face. The interviews were, focused on exploring participants’ attitudes towards barriers to achieving universal access to sexual and reproductive health. An interview was continued until the interviewer became sure she/he completely understood concepts provided by an interviewee. The mean duration of the KIIs was 75 minutes. Interviews were modified based on the former interviews. The questions were focused, among others, on the current state of the sexual and reproductive health in the health system, current barriers to improved quality of services and the challenges health providers faced in providing the sexual and reproductive health services. The first author conducted all individual interviews and moderated the focus group discussions. All interviews were audio-taped and transcribed verbatim on the same day (field notes). Transcripts served as the primary source of data for content analysis.

Three focus group discussions were conducted among health care providers. They were working in different fields of maternal health, HIV/AIDS, youth health, and family planning at different district and provincial levels. Another three focus group discussions were held among midwives working in different health facilities. The duration of discussions was between 41 and 78 minutes and the number of participants attending different focus group discussions varied from five to eleven persons. Focus group discussions were aimed to serve as a platform for obtaining a broad-spectrum of ideas on the sexual and reproductive health by looking at the topic from as many perspectives as possible. As such, we sought to capture as many features as possible of the shortcomings in achieving the goal of universal access to sexual and reproductive health.

All interviews were audio-taped and transcribed verbatim and qualitative content analysis was used for data analysis. The transcribed material was coded by the first author and discussed among all the co-authors to reach an agreement prior to detailed analysis. Similar codes were grouped to create categories and subcategories. A narrative approach is used to present the data.

Transcribed material were translated to English by authors and a bilingual Persian health care professional, (familiar with the topic under the investigation), and knowledgeable of the English-speaking culture, language-edited the text. A native English speaker with no knowledge of the topic under the investigation, then, translated the interviews back to Persian. The back-translations and the original texts were then collated and the English presentation modified accordingly by repeating forward and backward translation procedures until back-translations were deemed by all authors to be in harmony with the original transcripts.

### Ethical considerations

It is certified that all applicable institutional and governmental regulations concerning the ethical use of human volunteers were followed during this research. Informed written consent was obtained from all participants and the Ethical Committee of the Shahid Beheshti University of Medical Sciences as well as the Ethical Committee of the Ministry of Health & Medical Education approved the design of this study.

## Results

In this study interview and content analysis together yielded not only an enormous amount of data, but also rich data that could be examined for contextual implications. Several factors were found to play determining roles in preventing Iran from achieving universal access to sexual and reproductive health. The current obstacles can be modeled as follows.

### A cascade like presentation

Barriers could be generally looked upon from two perspectives of international and national levels. Local religious leaders have not actively attended international goal-setting programs. Therefore, they usually do not assume a positive attitude towards these goals. Such negative attitudes seem to be the most important factor hindering the progress towards universal access to SRH. Lack of international donors to fund sexual and reproductive health and sexual and reproductive and rights programs is also another barrier. In the national level both state and the society are interactively playing their roles. For presenting the cascade of obstacles at the state levels, we count barriers at each level from the strategic planning to implementation as follows:

A The international level

B The national level

a The State (Ethics)

I Strategic planning: adoption of international definition of the sexual and reproductive health targets and rights

II Strategic planning, mission: health vs. Rights

i Opportunities vs. Treats

ii Strengths vs. Weaknesses

iii Religious conservatism

III Policy-making

i Intended effects

ii Unintended effects

IV Legislation

i Resources allocation

ii Passing laws

V Execution

i Designing organizational structures

a Infrastructures and budget allocation

b Vertical approach

c Horizontal approach

ii Designing control systems

iii Matching organizational structures with control systems

b The Society (Morals)

I Attitude

i Gender role

ii Personal beliefs

iii Outcome evaluation

II Subjective norms

i Normative beliefs

ii Religious beliefs

## International level

The indicators of sexual and reproductive health have not been internationally agreed upon. The ideas and concerns of religious leaders have not been appropriately dealt with, nor clearly represented. Jurisprudents want the goal-setting process to be based on, stemming from, and made of religious law (Shari’a [ʃæri:’æ]).

“…Had the religious leaders actively participated in the process of goal-setting, they should’ve been more likely to live up their commitments to international conventions and treaties.”

“*… Are we expected to adopt pornographies on our TVs, or are we supposed to instruct our kids how to best get sexual pleasure? Are our children supposed to learn in schools how to be sexually attractive? When we are talking about sexual pleasure to our kids, aren’t we implicitly encouraging them to have a sexual experience? Or aren’t they getting more and more curious about exploring their sexual capacities? What is the threshold of age at which a kid is able to make a meticulous maneuver between the Scylla of the desire for exploring sexuality and Chary bids of morality? What is the international model? (A senior research associate).*”

“*We should respect people’s beliefs. Not all people all over the world can abide by instructions made on the basis of secularism and liberalism. There should be world-wide studies aimed at developing models that fit theocratic societies. Such an approach is conspicuously lacking* (A jurisprudent)*.*"

*“They have changed the world and now we are suffering from side-effects of irresponsible approaches. Sexual promiscuity, sexual deviation, sinful sexuality, sexual orgies are threatening our innocent children via internet or satellites TV channels. It’s a threat that we cannot tackle without international help. We believe that these threats are jeopardizing our children’s sexual security. We could not manage to find any intention in the relevant international plan of actions for putting an end to these processes* (A jurisprudent)*.*”

## National level

### State

#### Strategic planning: adopting international definition of the sexual and reproductive rights targets and rights

We need to either develop our own yard stick or adopt internationally-developed indicators [54] to measure universal access to the sexual and reproductive health and rights.

#### Strategic planning: right-based approach vs. Health-based approach

The current strategies were found by participants to be out-of-date; they insisted that alternative, up-to-date, comprehensive, client-oriented, efficient methods must be sought. It was suggested that the role of husbands, peers, parents and community needs to be accounted for in designing and implementing programs. As a strategic approach to the universal access to the sexual and reproductive health targets and rights the issue must be pursued in the context of health rather than rights. Inasmuch as the public seeks freedom and then individual states contradictorily seek standards (and, thus, the authority for implementing those standards), a paradigm shift is imperative for strategic planning. Rights are matters of entitlement, which frees people of authoritarianism. The state considers entitlement of each and any right as a threat to its authority.

“… The society is not currently prepared to accept the sexual rights. Nonetheless the issues could possibly be discussed from the perspective of health (A top executive of the Ministry of Health and Education).”

*“Let’s have a look on the family planning in which sexual and reproductive health was horizontally integrated, say like a Trojan horse. Many sexual educations have also been done during our struggling efforts to tackle AIDS/HIV. I mean the sexual educations should be indirect and implicit rather than direct and explicit* (A provincial level manger)*.”*

“Islam is believed to have many instructions for sexual and reproductive health. …What goes in opposite direction with Islam is the “rights” that embodies freedom of individuals to decide on their sexual and reproductive behavior. This freedom brings the Islam’s standards into challenge.”

Participants pointed out that Iran’s current political atmosphere does not allow issues like “adolescents’ sexual and reproductive health and sexual and reproductive rights” to be extensively discussed.

“Talking about taboos can weaken political position of politician; they might not risk it, thus.”(National level manager)

### Public level obstacles

The most difficult and challenging targets are adolescents’ fertility rates and unmet needs for family planning. The former, deals with the sexual education to adolescents-an extremely sensitive issue; and the latter points to safe abortion. Meeting unmet need for family planning will reduce the rate of resorting to abortion by preventing unwanted pregnancies. Furthermore, avoiding unwanted births (once there is a pregnancy) requires unveiling the restrictions imposed on abortion (according to national decisions; e.g., in the case of: rape or incest; fetal abnormality; risk to the life of the mother; or, to the health of the mother; economic and social decisions; etc.) and ensuring its safety under the permitted conditions.

“Based on the indicators defined by MDGs targets, a major part of the job regarding sexual and reproductive health has already been done. Few jobs (!) remain to be accomplished; poignantly the most difficult and challenging ones, though. .. That’s the adolescents’ fertility rates and unmet need for family planning. The former, deals with the sexual education to adolescents-extremely tender, untouchable zone. The latter implicitly conveys the issue of safe abortion-no man’s land, I would say. …”

“…The sexual education has long been looked upon as a recreational activity that can potentially end up in the increase in sexual promiscuity; this is not unique to our society…”

“…There are worse things than giving education about sexual health to our kids. It’s far worse to see pregnancies among adolescents who are unprepared, unable, and unwilling to put in the time and effort it takes to be good parents; to see adolescents contracting and spreading STDs/AIDS/HIV. …”

#### Religious conservatism

Sexual health services have generally been neglected because providing them requires governments to acknowledge sexual rights to sexual pleasure and sexual orientation, and to address issues such as gender roles and power imbalances within relationships.

*“Islam is believed to have many instructions for sexual and reproductive health. …What goes in opposite direction with Islam is the “rights” that embodies freedom of individuals to decide on their sexual and reproductive behavior. This freedom brings the Islam’s standards into challenge. Furthermore, Shiite notion of “the rule of the jurist (Velayat Faqih,* [wiläjæt fæɣiːh^ə^]*)” places Shiite religious leader (Mojtahed,* [mudʒtæhid]*) on an exceptional position of getting involved on policy-making …. However, what enables us to …improve our law system… is the dynamism of Ejtehad* [idʒ^ə^tihad] *…that is to make new interpretation on the religious basis i.e.* (a religious leader)*.”*

*“The role of the Expediency Discernment Council of the System could not be overemphasized. …this enables us to augment the traditional Islamic rules so that we can efficiently handle problems encountered in the modern and post-modern era* (A top executive of the Ministry of Health and Education).*”*

#### Legislation and execution

##### Designing organizational structures

All interviewees consistently asserted that the health system in general and the reproductive health in particular lack effective approaches to health promotion and prevention. All participants pointed out that the root and social determinants of reproductive health should be dealt with beyond the territory of Ministry of Health and Education.

*“We don’t know where to place the sexual and reproductive health services. We don’t know how to organize the services. Policy-makers need to decide between inserting vertical sexual and reproductive health programs (stand-alone) into the health system and horizontally integrating them into other elements of health system. The final policy could be a trade-off between the two* (A national level policy-maker).*”*

The poor coordination and collaboration between different levels, ties and directorates were issues that were frequently introduced during interviews. There was found to be a need for bridging the gaps between the separate programs. Programs such as maternal health, family planning, and youth health continue to lack a productive interaction or integration.

*‘In the Ministry of Health, all programs are sectorial and there is no sharing or harmony between them (those who hold authority in the field) to address the needs of population. There are departments with different titles say “Control of HIV/AID” or “Population Management,” or “Family Planning” with all sharing the concept of sexual and reproductive health. Meanwhile, all programs have failed to appreciate or deal with interlocking issues like abortion or high-risk behaviors of adolescents. They should all be working to address these issues not only one department* (A senior academic professor).”

It was repeatedly mentioned during interviews that one of the key challenges in health delivery system is the poor organization of the public and private sectors. The majority of people living in urban areas seek services from private sector where health providers deliver sexual and reproductive health services with varying content without any standardized protocol. The services delivered in the private sector are not recorded (documented) nor reported; and thus, are seldom realistically monitored and evaluated.

“*Our system is fragmented, some health care providers provide health care services on their own as they see fit. They are unwilling to refer the clients even if they feel they are not competent enough. Even worse, it comes barely to notions of many of the providers that they might not be scientifically competent or have necessary experience for delivering certain health care services. There are also many organizations in parallel to each with no synergy. In this situation, we are wasting our resources* (A provincial level manager)*.*”

*“The main problem in our system is that many of patients affected with sexually transmitted diseases seek services in the private sector and frequently have been observed not to be referred for harm-reduction behavior consult* (A national level manager).”

#### Economy and infrastructures

Governments do not have the capacity to provide universal access to sexual and reproductive health. Human resources (trained doctors, nurses and midwives) are not enough for providing services; supplies of drugs and contraceptives are often unsettled; and there is a lack of technical expertise in some areas. Poor communications and transport infrastructure can prevent access to services in rural areas, especially in maternal health care where transport to referral services with adequate facilities is an essential component of dealing with emergencies.

*“Many women in some regions have poor health and many of them die during pregnancy and childbirth because most of them are illiterate or poor […]. To eliminate maternal mortality we need to revise our programs and address these issues* (A national level manager)*.”*

##### Policy-making: policy ambiguity

While religious leaders participating in this study highlighted the importance of the reproductive health of adolescents, they pointed out that lack of a clear comprehensible language to communicate reproductive rights is a critical barrier to provide reproductive health services for adolescents and unmarried young people. They also mentioned that a clear strategy is lacking to guide policy-makers on how the sexual and reproductive health services are to be supported by the government.

“It’s yet to be recognized that sexual health is distinct from reproductive health and that the need for sexual health services and information are going beyond those concerning reproduction and HIV.”

Standards can be established for minimum acceptable care accessible to everyone; for instance, timely access to necessary care and numbers of doctors to be trained to serve disadvantaged population subgroups [55].

“National laws concerning sexual and reproductive health often remain ambiguous and inconsistent. For example, while 12 year olds girls are legally capable of consenting to marriage, we are not providing them with sexual education. Such ambiguities can provide a foundation upon which service providers use their discretion and restrict access to some groups of people, based on personal prejudices (A national level manager).”

##### Policy-making: client-centered approach

When evaluating services, improving quality of care requires that patients’ perspectives and levels of satisfaction are taken into account and incorporated into policy decisions. This means that in addition to clinical factors, providers need to be aware of their patients’ cultural values, social concerns and individual needs. Patients usually determine quality of care based on: acceptable waiting times, convenient opening hours, confidential relationships, availability of gender-sensitive services, continuity of services, choice of contraceptive method, and being treated with dignity and respect.

*“Any plan to achieve not-yet-achieved targets on sexual and reproductive health and sexual and reproductive rights must be client-centered. We’ve got to first deal with perceived needs of clients (i.e. the needs perceived by clients as needs). Otherwise they might not want them* (a national level policy maker)*.”*

##### Resource allocation: sustainable financing

To achieve universal access, it is essential for sexual and reproductive health services to be affordable even for the poorest people in societies. In many instances, this means that services must be free. Reductions in donor funding mean that providing free services is becoming increasingly difficult to sustain, especially in countries with limited resources.

##### Resource allocationhuman resources

The majority of the respondents pointed out that inadequate human resource capacity is a key challenge to improving access to reproductive health care services. They also believed that the accessibility and quality of reproductive health care services have been affected by specialization of training of human resources. While primary health care providers are generally available in health care centers free of charge, there is inadequate focus on training multitask health care providers working in the Health Care Network.

*“We need to find the target population that are most likely to benefit from services … We also need to define the most important and critical services that should be delivered first. … There are many resources we can utilize and mobilize e.g. mosques, municipalities health houses, schools, and religious teachers, health instructors, non-scientific instructors, universities, sex clinics, 20 000 health centers all over the country, ministry of Islamic culture and guidance. Books, Videos, and Lectures must be taken into account while we are making policies* (A national level policy-maker).*”*

*“Some components of the sexual and reproductive health are critical and should be dealt with urgently. As such, they should be given the highest priority while allocating recourses … Surprisingly, these dimensions, are not controversial. In fact, they could be shown to have already been agreed upon; for example sexual violence, rape, and young-age marriage. These are subjects that dealing with them may not insult public purity and can be easily pursued without any resistance … (*A religious leader)*.”*

#### Operational level

##### Diverse high-quality services

Perceived quality of care is an important factor that determines whether people choose to utilize sexual and reproductive health care services. Evidence from Bangladesh, Senegal and Tanzania suggests that in areas where women felt that they were receiving a high standard of care, they were more likely to use contraceptives than in areas with lower quality health facilities [56].

*“It is imperative to improve quality of care … expert health care providers, skilled consultants, safe procedures, accurate information and reliable products, choice of contraceptive method and so on …(*A national levels manager)*.”*

##### Education

Iran’s health system has a unique structure with the medical education having been integrated into Ministry of Health in order to better provide health care services to the community. However, there is no policy to upgrade medical education addressing emerging issues such as sexual and reproductive health education to adolescents. Participants pointed out that the current medical curricula are mostly disease-oriented; focusing on specialized treatment and secondary prevention services. Inter-disciplinary, community-based approaches are conspicuously lacking.

*“In recent years, the medical education system has been more involved with training specialists and sub-specialists while neglecting a deep need for multi-task health care providers. Midwives can easily promote a broad spectrum of reproductive health services including “safe motherhood” and “pre-marital counseling.” Nonetheless, midwives have barely been mobilized towards these targets* (Manager)."

*“We know that the core of family planning services is counseling but we have too little time to counsel the clients. You can see too many clients waiting in health centers and a midwife has to examine them with no time to record her findings. We need more health care providers* (Midwives, urban health center, FGDs).”

*“Our physicians don’t know how to educate and consult clients. It is said that the basis of education is proper communication. Well, medical students are generally not instructed how to communicate properly. There is no curriculum such as communication skills in medical schools* (Health policymaker).”

The national program provides a formal family planning education package for the higher education students and develops the initiative of a mandatory pre-marriage course for newlywed couples [5,19]. However, these programs do not adequately cover what young people need to know about sexual and reproductive health [5]. Many responded suggested that these programs should be implemented more widely with stronger emphasis on sexual health promotion, but the key challenge to expanding these services is the inadequate capacities of skilled health care providers.

*“We lack trained expert personnel who can provide a comprehensive consult or for their clients (couples). … Training such skilled health care providers calls for a great deal of energy (*A provincial level manager).”

“The service providers’ attitude is a critical factor in determining whether or not an adolescent seek sexual and reproductive health services. Stigmatization and discrimination against adolescents (stereotyping or mislabeling) is a common experience.”

### Society (morals)

There are a number of interlocking social and cultural factors reinforced by restrictive laws and policies. These factors can impede access to services and information. People who are most vulnerable to sexual and reproductive ill-health are those who have been most extensively deprived from sexual and reproductive health services.

#### Religion and opportunities

Religion was found to be the strongest determinant factor in achieving sexual and reproductive health targets and entitlement or confinement of sexual and reproductive rights.

*“Ethics emerge from value conflicts. People value both health and religion; however, the health is usually valued higher than the religion. For example people are allowed to deny their faith to save their lives* (A national level manager).*”*

#### Social taboos

Issues around sex and sexuality are taboo in Iran and other related cultures. As such, perceived stigma and embarrassment make people reluctant to discuss and address sexual health issues. Taboos are even more pronounced for people who do not conform to socially accepted norms.

*“Unmarried adolescent girls are vulnerable to violence and sexual abuse. They are also susceptible to the consequences of early unplanned sexual experiences including unwanted pregnancy, STDs, and unsafe abortions. Nonetheless, they are frequently observed to be deprived of or have limited access to sexual and reproductive health services.* (A national level manager).*”*

#### Gender role

The society is “mard-salar [mærd-sälär]” i.e. gender norms in Iran tend to make men macho. That is the society marginalizes women, renders them passive, and thus makes all of them vulnerable to sexual and reproductive problems while restricting their access to the sexual and reproductive health services.

*“Men may associate masculinity with taking risks in their sexual relations; this exposes them to HIV and STIs. What makes the situation even more complicated is that they may be reluctant or too embarrassed to seek out appropriate health information and care. These are often focused on women* (A provincial level manager)*.”*

#### Knowledge

Educational status was mentioned by participants to have impacted the way people perceive the sexual and reproductive health.

*“Many of the adults don’t even know how to name their sex organs. Kids are instructed to name their eyes, ears, hands, feet, but they do not know how to name their “?”… Maybe we should start with parents* (A professor).*”*

#### Norms

Cultural value of being conservative was found to have a significant impact on health-seeking behavior. The fear of being stigmatized as an individual who deviates from societal norms is strong. This finding was closely associated with gender norms ascribed to women, namely that they should remain “virgin”, sexually inactive before marriage.

*“Norms and cultures being deeply rooted in religion have their exceptional characteristics in Iran. There are many normative beliefs that may facilitate entitlement of the sexual and reproductive rights.”* (A national level executive)

#### Strengthening participation and accountability

Active involvement of marginalized clients in decision making processes at all levels, and providing them with the opportunity to hold service providers and policy makers accountable for discriminatory practices, corruption, or poor quality services, helps to redress inequalities in access to sexual and reproductive health services and ensure that they are acceptable and appropriate [57].

*“Accountability mechanisms are required to ensure an acceptable quality of service. This opportunity is lacking* (A provincial level manager)*.”*

## Discussion

The barriers to the universal access to the sexual and reproductive health and sexual and reproductive rights (as recognized by MDG target 5B) were examined using a qualitative approach. A gap does exist between ultimate targets and current achievements. The gap could be looked upon from two perspectives. One denotes what the health system could achieve but it would not (e.g. safe abortion or disparities based on age, sex, and marital status); and the other denotes what the health system would achieve but it could not (e.g. disparities based on residency status). The former presents how far Iran is from the goals advocated by MDG. The other gap presents patchy, uneven distribution of the achievements. According to the recent trends observed in the indicators of the sexual and reproductive health, “unmet need for family planning” is the goal that deserves special attention. Unmet need for family planning could directly be translated to unwanted pregnancies and unwanted childbirth. The former calls for sexual education to people including adolescents and the latter for safe abortion.

### Lack of political commitment: when there’s a will there’s a way

Investing in “sexual-and-reproductive-health-for-all” requires tackling constraints at many levels: in the household and community, within the health-service delivery system, at the health-sector level, in broader policies and public institutions, as well as among donors and international organizations. The quality of governance at national and local levels is crucial. Good governance means that governments have the commitment, credibility and capacity to devise and implement sound policies, which directly affect the potential for strengthening health systems and for creating an environment that makes universal access to them possible [58].

Fathalla and colleagues argued that universal access to sexual and reproductive health is achievable but we need to create the necessary environment for the implementation of appropriate programs and, above all, both nationally and internationally, we need the political will and the determination to act [59].

Tackling sexual and reproductive ill-health requires courage and integrity. Sexual behaviors, attitudes, and norms vary around the world. There is no one-size-fits-all solution that will reduce risky behavior. Politicians, religious leaders, and bureaucrats have to decide that women’s lives and rights are worthwhile and not challenging to their authority [5].

### Weakened health system and lack of skilled health care providers

Sexual and reproductive health care needs to be inserted into a strong health system. Such a system is the one that is adequately funded, relies on well-regulated private-public partnerships, is effectively governed, and has insurance and other mechanisms to ensure healthcare coverage for all. It also has the infrastructure essential for operating effectively, efficient logistics to ensure a steady supply of commodities, clear guidelines and policies, and sufficient numbers of providers who are well-trained, culturally rooted, and adequately compensated. As presented here, a fundamental issue is the number of multitask health care providers. But coverage can be achieved by better use of existing personnel. Furthermore, structural adjustment processes have often led to cuts in health-sector spending, privatization of services, and the introduction of fees into public healthcare. These changes have raised the barriers to healthcare for many, especially the poor and other marginalized groups.

### Trends in funds

Lack of political will has been translated to a corresponding lack of financial commitment to sexual and reproductive health (excluding HIV) by both international donors and national governments. The proportion of donor funding has been reduced in different areas of the sexual and reproductive health, in particular family planning.

To some degree, reproductive health has been a victim of its own success in lowering aggregate fertility rates. Some have concluded that population growth is no longer a serious problem. *“Iran’s family planning program has been lauded as an ‘Iranian Miracle’ and modeled around the world, including in the US. It holds the record for the largest and fastest decline in fertility ever. The total fertility rate (TFR) dropped from 6 children per woman in the mid-1980s to 2.1 children per woman in 2000. This greatly exceeded expectations; the TFR in 2000 was less than half of what had been planned for 2011. "It confounded all conventional wisdom that it could happen in one of the world’s few Islamic nations (Jalal Abbasi-Shavazi, a demographer at the University of Tehran)*[45,60]*.”*

What is not usually recognized is that poor people are still experiencing high unintended fertility rates, which contributes to their persistent poverty [45]. In a major reversal of once far-reaching family planning policies, the budget for the population control plan had been completely removed (Iran Daily Brief, 3 August 2012) in an attempt to avoid an aging demographic similar to many Western countries that are struggling to keep up with state medical and social security costs. Although the government has not outlawed contraception outright and it should remain available through the private sector, cutting back services will create serious implications for lower income women who rely on public services.

### Opposition to aspects of sexual and reproductive health

Conservative trends present challenges to advancing sexual and reproductive health and reproductive rights. Women’s control over their own sexuality and reproduction can be perceived as a threat to religion, decency, and the dominant patriarchal social order, especially when sought out-of-wedlock. Religious interpretations are used to justify restrictions on sexual and reproductive health education and services, especially sexuality education and services for adolescents, and safe abortion (where not against the law).

### Operational impediments

Considering the scope and complexity of sexual and reproductive health issues, we need a nationally agreed upon medical, social, moral, legal, and operational definitions of the sexual and reproductive health and reproductive rights.

Accelerating universal access to sexual and reproductive health requires urgent and sustained action by the global community. We need adequate funding, firm political commitment, courageous and creative programming, and the involvement of diverse actors, including faith-based, civil society, and private sector partners.

#### Organizing a program for achieving universal access to sexual and reproductive health

The structure of the system through which to deliver sexual and reproduction health has yet to be determined.

##### Vertical vs. horizontal approach

In vertical approaches, interventions are provided through delivery systems that typically have separate administration and budgets, with varied structural, funding, and operational integration with the wider health system. In the horizontal approaches (integrated model), services do not have separate administration or budgets and are typically delivered through health facilities that provide routine or general health services [42]. The benefits of vertical programs are that they focus on the population need for a particular disease, use specialist staffs (who generally manage just one condition), have educated resources, and operate in a project mode with clear objectives to be achieved in defined time scales. Consequently, it is suggested that they tend to be more efficient than horizontal approaches in achieving objectives. In contrast, horizontal approaches focus on the individual, use generalist personnel who deal with multiple symptoms and conditions, respond to user needs as well as demand and are more holistic in scope, often with inter- and intra-sectoral links [42].

##### Integrating sexual and reproductive health into primary healthcare

The ICPD, as a key thrust, called for integrating sexual and reproductive health into primary healthcare. Since then, it has been exceedingly recognized that health systems need to be strengthened overall and as a prerequisite for achieving universal access to reproductive health. Achieving universal access to reproductive health means taking measures to eradicate inequities by eliminating barriers to access, whether they are economic, structural, or cultural. It requires government action, usually a mix of financing, legislation, and regulatory mechanisms that help to ensure universal access and good quality services [61].

##### Integration to HIV/AIDS

If sexual and reproductive health policies and programs are developed and implemented in isolation, universal access will not be achieved. Rather, they could and should be integrated with the prevention of HIV [62].

The current sexual health programs mainly focus on HIV/ STI and neglect the promotion of broader sexual health of women, men, and adolescence. Since the arrival of the HIV/AIDS pandemic, Iran has formulated the National HIV/AIDS Control Programs (2002). The health system has been providing integrated consulting and care services. These services are provided through consulting centers for behavioral disease (HIV/AIDS and Substance Abuse). All services, including provision of anti-retroviral therapy to people living with HIV, are free in these centers. Iran has been a leading country in HIV prevention and treatment in the Middle East; and HIV transmission among injecting drug users has reduced significantly [63].

Young people in Iran are a major group at risk of HIV/STI. Because of an increase in the age at first marriage [64], an increasing proportion of young people are potentially engaged in risky premarital sexual activities [65]. However, large numbers of Iranian young people lack information about safe sex [66,67]. This may be affected by culture, governmental, and financial policies.

### Adolescents’ reproductive health

In Cape Town, 2009, the International Federation of Gynecology and Obstetrics (FIGO) identified 3 issues needing urgent attention:

1 Adolescent sexual and reproductive health;

2 Unsafe abortions and related mortality and morbidity; and

3 HIV prevention and care [55,68,69].

The critical gaps identified included:

1 The lack of information on sexual and reproductive health issues for adolescents, such as safe sexual practices, contraception, risks related to early childbearing, unsafe abortion and its adverse consequences.

2 Inadequate linkages between sexual and reproductive health and HIV interventions that result in missed opportunities for addressing both [55,70-76].

Recommendations included the use of innovative information dissemination techniques, ensuring access to family planning and comprehensive abortion care to the full extent allowed by national laws, in accordance with FIGO and WHO guidelines, and promotion of universal HIV counseling and testing with opt-out strategies within sexual and reproductive health services and information on sexual and reproductive health in all HIV services [59,77].

There are few studies on unmet needs to be specifically conducted among adolescents or to compare the situation among adolescent and adult girls. However, the multivariable adjusted odds ratio for having unmet reproductive needs has been shown to decrease by ratio of 0.95 (95% CIs: 0.94-0.97) for a 1-year increase in age [47].

The age at first marriage has been increasing with no evidence being available to indicate that the trend is leveling off or reversible [44]. On the other hand less than 50% of adolescents are currently holding strong attitudes against having pre-marital sex. The mean age at sexual debut has been reported to be 15 years for boys and 20 years for girls [78]. Furthermore the access to satellite TVs has been reported to be a contributory factor [79,80]. It is not surprising, thus, to see the premarital sexual debut paralleling the increase access to satellite TVs.

According to the Population Action International (2002), Iran had implemented national governmental programs on adolescents sexual and reproductive health including compulsory premarital counseling programs for all couples contemplating marriage [81]. Although Iran agreed to teach adolescents health and even sexual health as a result of negotiation between academics and religious leaders in New York during ICPD events in 1999 (provided that its content is appropriate for a student’s age and is pursued under parental supervision) [26], there continues to be no formal reproductive and sexual health education in schools, health units, and even amongst families, because such programs for young students remain taboo. There is a general belief that sexual education may end up promoting sexual promiscuity. But evidence base is uncertain. In the absence of strong and high-coverage programs, adolescents are at risk of STDs, HIV/AIDS, and unwanted pregnancies [81-83].

A number of educational programs have been developed to provide information on adolescent health, family planning, STD, HIV/AIDS, and even sex issues to girls and boys taking their age into account. These programs have been welcomed by families and the authorities [26]. Shirpak et al. have recently demonstrated that even in the societies where peoples’ religious and cultural background might seem to make sexual education an impossible task, choosing the educational content based on the target group’s needs and cultural and religious background can pave the road to success [84]. Due to the sensitivity of adolescent reproductive health issue, efforts are being delegated to formal and informal education channels such as Parents/Teacher Associations. This channel seems to be appropriate and culturally accepted, though it should be further strengthened.

In practice, representation of adolescents in the planning processes for sexual and reproductive health services has been limited. The participation is commonly observed to be restricted to service delivery, and was not extended to the design of policies, legislation, and allocation of budgets. Adolescents are not consulted as much as mainstream health organizations. This may have its roots in the fact that even within the forums for participation, they lack the skills, information, or representation to have a voice among more powerful participants [85].

An examination of Iran’s policies on family planning, women’s education, and personal status law demonstrates the flexibility and sophistication in co-opting competing ideologies. In the process, the religious political leaders have revised many of their Islamic stances, particularly on the issue of family planning [86].

### Implications of the universal access to reproductive health

Creating an effective policy framework for sexual and reproductive health involves the establishment of relevant national laws. Equally important is setting out regulations, norms and guidelines that make implementation of those laws feasible and effective.

#### From family planning to sexual and reproductive health: paradigm shift

International Women's Health Coalition have addressed the need to transform population policies to account for development and human rights concerns and to transform family planning into sexual and reproductive health services that advance health and rights, not simply the achievement of demographic objectives [87].

#### Religious-based strategy planning: rights-based approach vs. Health-based approach

Dixon-Muller et al. have delineated five dimensions of sexual behavior:

1 Sexual relationships and the right to choose one’s partner;

2 Sexual expression and the right to seek pleasure;

3 Sexual consequences and the right to cooperation from one’s partner;

4 Sexual harm and the right to protection; and

5 Sexual health and the right to information, education and health services [88,89].

Inasmuch as ethnicity and religion continue to be strong determining factors, presuming the atmosphere of neutrality is very important. Negotiators should not be judgmental while encountering religious leaders. Sensitive issues are best addressed in the context of health. The acceptance is more likely to be granted to technical or scientific approaches. At the beginning of a project, as much time as necessary must be invested on clarifying the subject of interest and ambiguity that might arise should be given due patience and energy it takes to be resolved.

“Rights” always implies the capacity to make autonomous decisions, to assume responsibilities, and to fulfill needs in both the individual and the collective sense. The construction of rights implies the re-balancing of power relations and a horizon of justice [90]. At Cairo debates became heated, as many delegates adopted rights-based approaches promoting the ideas of sexual and reproductive health and women’s empowerment. Roman Catholics, conservative Christians, and Muslims were concerned that, whether explicitly and/or implicitly, feminists and liberals were arguing for women’s right to abortion. This area of contention resurfaced throughout the process of drafting the MDGs [91-93].

#### Unsafe abortion

There is nothing, in MDG 5 specific to the prevention of unsafe abortion and it continues to be a “Missing Link in Global Efforts to Improve Maternal Health [94-96].” However, World Health Organization (WHO) deems unsafe abortion one of the easiest preventable causes of maternal mortality and a staggering public health issue. Carol Smithyes argue that:

“*MDG 5 will not be met unless the burden of mortality from unsafe abortion is addressed. MDG 5 requires a 75 per cent reduction in the maternal mortality ratio (MMR) by 2015 and some key countries, which will determine the overall achievement of this MDG, are seriously off track in meeting this target. Unsafe abortion is the second leading cause of maternal mortality worldwide, and in some countries more than a third of maternal deaths are due to post-abortion complications*[97].”

FIGO in its endeavor to assist nations toward achieving universal access to comprehensive sexual and reproductive health services prioritized unsafe abortion as one of the entry points toward improving women’s health and reducing maternal mortality and morbidity [98]. Unsafe abortion accounts for 13% of maternal mortality and results annually in nearly 70, 000 deaths worldwide; 99% of these occur in low-resource countries with only a few in high-resource regions of the world [61,99-101]. Besides mortality, unsafe abortion can be responsible for temporary or permanent disability, including secondary infertility, in millions of women [68].

Young women and adolescent girls die every day from preventable pregnancy and childbirth-related causes because they are marginalized, because their human rights are violated, and because their needs are ignored, particularly their sexual and reproductive health needs. They are poorly targeted by our national policies and programs. In fact the programs are often based one-size-fits-all strategies not consistent with their realities; even these programs are most of time absent in our budget allocations.

Our laws on age at marriage need to be modified, to create mechanisms to enforce these laws and effectively implement them. Adolescent girls and young women need to be equipped with all the information necessary for having healthy sexual and reproductive lives. This should be provided to all adolescents and young women, in and out-of-school, in the cities, the rural, and the remote areas. The structural barriers should be eliminated: laws, facilities’ coverage, and user fees that prevent adolescent girls and young women from accessing comprehensive sexual and reproductive health services. This must include life-saving contraceptives, with no restrictions. Laws, infrastructures, and services need to be in line with our people’s realities, and adolescent girls and young women’s needs must be fulfilled, regardless of age, economic, or marital status.

Unsafe abortion should be introduced to policy-makers; they should recognize that this is not a politically insensitive subject to censor, but a reality that kills in silence. They should recognize that 70 000 women’s deaths each year (half of which occurring among young women) can be avoided by ensuring access to safe abortion services, as provided in the World Health Organization’s publication Packages of Interventions for Family Planning, Safe Abortion Care, Maternal, Newborn and Child Health [102].

### Limitations

The views of the key interviewees of current study may not be representative of all experts in Tehran or in Iran.

## Conclusion

The barriers to universal access to sexual and reproductive health and sexual and reproductive rights (as recognized by MDG target 5B) were examined using a qualitative approach. A gap does exist between ultimate targets and current achievements. The gap could be looked upon from two perspectives. One denotes what the health system could achieve but it would not (e.g. safe abortion or disparities based on age, sex, and marital status); and the other denotes what the health system would achieve but it could not (e.g. disparities based on residency status). The former presents how far Iran is from the goals advocated by MDG; whereas, the other gap presents patchy, uneven distribution of the achievements. According to the recent trends observed in the indicators of the sexual and reproductive health, “unmet need for family planning” is the goal that deserves special attention. Unmet need for family planning could directly be translated to unwanted pregnancies and unwanted child births. The former calls for sexual education to people including adolescents and the latter for safe abortion. Barriers could also be looked upon from two perspectives of the international and national standpoint. Religious leaders not actively attending international goal-setting programs seems to be the most important factor hindering the progress towards universal access to the sexual and reproductive health. Lack of international funds for sexual and reproductive health and sexual and reproductive rights programs is also another barrier. In national levels, both state and the society are interactively playing their roles. For presenting the cascade of barriers at the state levels, we count barriers at each level from the strategic planning to the implementation. The state’s decision not to fund public services is a barrier that is worsened by low levels of international support. This should be added to the poor organization and lack of human resources in the field of sexual and reproductive health. At the civil society level, social and cultural factors should be considered as background to all stages of state- and international-level challenges.

## Abbreviations

FGD: Focus group discussion; FP: Family planning; KIIs: Key informant interviews; MCH: Maternal and child health; MOHME: Ministry of Health and Medical Education; NA: One of the authors; NGO: Non-governmental organization; PHC: Primary health care; PoA: Plan of action; SRH: Sexual and reproductive health; STI: Sexually transmitted infection.

## Competing interests

The authors declare that they have no competing interest.

## Authors’ contributions

NA, AR and MP contributed to the conception and design of the study. NA conducted interviews with key interviewees and undertook data coding and data analysis with oversight from AR and MP and NA drafted the manuscript. All authors read and approved the final manuscript.

## Pre-publication history

The pre-publication history for this paper can be accessed here:

http://www.biomedcentral.com/1472-6963/13/241/prepub
